# Application of Bioorganic Fertilizer on *Panax notoginseng* Improves Plant Growth by Altering the Rhizosphere Microbiome Structure and Metabolism

**DOI:** 10.3390/microorganisms10020275

**Published:** 2022-01-25

**Authors:** Rui Shi, Shu Wang, Bingjie Xiong, Haiyan Gu, Huiling Wang, Chao Ji, Weijia Jia, Abraham Rami Horowitz, Wenjie Zhen, Jiftah Ben Asher, Xiahong He

**Affiliations:** 1 Key Laboratory for Forest Resources Conservation and Utilization in the Southwest Mountains of China, Ministry of Education, International Ecological Foresty Research Center of Kunming, College of Horticulture and Landscape Architecture, Southwest Forestry University, Kunming, 650224, China; shirui@swfu.edu.cn (R.S.); wangshuswfu@outlook.com (S.W.); xiong18511478285@163.com (B.X.); guhaiyan@swfu.edu.cn (H.G.); jwj0618202106@163.com (W.J.); 2 State Key Laboratory for Conservation and Utilization of Bio-Resources in Yunnan, Yunnan Agricultural University, Kunming 650201, China; whuiling1018@126.com (H.W.); jichao1100@163.com (C.J.); 3 Laboratory for Quality Control and Traceability of Food, Tianjin Normal University, Tianjin 300387, China; hrami@volcani.gov.il; 4 The Katif R & D Center, Ministry of Science and Technology, Sedot Negev Regional Council, Negev 84965, Israel

**Keywords:** Sanqi ginseng, bioorganic fertilizer, bacterial diversity, community composition, soil metabolism

## Abstract

Bioorganic fertilizers can alleviate (a) biotic stresses and sustainably increase crop yields. The effect of bioorganic fertilizers on the rhizosphere bacterial community of *Panax notoginseng* and soil metabolism remains unknown. Here, we tracked the changes in the soil physicochemical properties, bacterial microbiota responses, and soil metabolic functions after the addition of a bioorganic fertilizer in a *P. notoginseng* field. The application of a bioorganic fertilizer reduced the soil acidification, improved the organic matter, and increased the contents of the total/available soil nutrients. Soil amendment with a bioorganic fertilizer significantly affected the structure of the rhizosphere bacterial community, leading to the enrichment of specific bacterial consortia such as *Rhodanobacter*, *Arthrobacter*, *Sphingomonas*, *Devosia*, *Pseudolabrys*, *Luteimonas*, *Lysobacter*, *Nitrosospira,* and *Nakamurella*. Previously, many of these genera have been associated with nutrient cycling, plant productivity, and disease suppression. Metabolome analysis further highlighted that the bioorganic fertilizer treatment significantly reduced phenolic acids and flavonoids and enhanced organic acids, saccharides and alcohols, and amino acids. This result indicates a high survival of bacterial microbiota in the rhizosphere and an availability of nutrients for *P. notoginseng* growth. This work showed that the application of bioorganic fertilizers significantly improves soil health status, alters soil metabolic functions, and stimulates a specific subset of rhizosphere microbiota for nutrient cycling and disease protection in *P. notoginseng*.

## 1. Introduction

Soil microorganisms are continuously engaged in several ecosystem services, including decomposition of organic matter, nutrient cycling, plant growth, and disease protection [1,2,3,4]. At a system level, a stable and specific soil microbiome structure predominately determines the agricultural land’s positive ecological functions and productivity [5,6,7]. The coevolutionary dynamics between members of soil microbiota can be disrupted by agricultural management strategies such as crop rotation, pesticide use, tillage, and chemical fertilizer, leading to the change in the taxonomic and functional profile of the soil microbiome [7,8]. Over the past decades, farmers have amended agricultural field soils with significantly high amounts of chemical fertilizers, which have reduced soil pH and changed the microbiome composition [9,10,11]. Excessive application of chemical fertilizers has been recently hypothesized as suppressive for the development of sustainable agriculture [12]. As such, replacing chemical fertilizers with organic fertilizers is a prerequisite for positively influencing soil health and microbial biomass in order to enhance crop productivity [12,13].

Sanqi ginseng (*Panax notoginseng* (Burk.) F. H. Chen), belonging to the family Araliaceae, is reported as a valuable medicinal plant with many benefits for human health [14]. In China, *P. notoginseng* has been cultivated for more than 200 years. Its roots are widely used in herbal medicines for health purposes, including, but not limited to, curing cancers, cardiovascular diseases, and coronary artery diseases [15,16]. It is also currently listed as a dietary food supplement by the United States Dietary Supplement Health and Education Act [17,18]. In recent times, the cultivation of Sanqi ginseng has been increased many folds to meet the consumers’ demand. Much of the research has recently focused on improving the yield of this vital crop by adopting agricultural management strategies. *P. notoginseng* generally grows under the shades of forests without tillage at the slopes of mountains. However, farmers can produce Sanqi ginseng under artificial shade structures in field conditions at high densities in order to achieve a maximum profit [19]. According to a field survey, the soil nutrients such as total and available nitrogen, total and available phosphorus, and organic matter content were significantly higher in one-year-old *P. notoginseng* soils than non-planted soils [20]. Excessive application of fertilizers affected soil nutrient imbalance that leads to nitrate contamination and has been shown to influence the flavonoids, glutathione, anthocyanin, ascorbic acid, and antioxidant activities in *Labisia pumila* [21], apple [22], and basil [23]. High N content in the soil also significantly disrupts the microbial diversity, and a low microbial population often found in soils is conducive for disease development [24]. In this situation, *P. notoginseng* has been already reported to be attacked by many soil-borne pathogens causing root rot, black spot, round spot, and gray mold diseases [25,26]. A reasonable and optimal soil fertilization strategy that improves the growth of *P. notoginseng* and protects the plants from pathogen invasion is urgently required to obtain a high yield.

Bioorganic fertilizers are rich in organic matter and biological microorganisms and tend to have a positive impact on microbial community structure than chemical fertilizers. Organic fertilizers in the form of animal manure, composted organic matter, and plant residues can alter the structure and functions of the soil microbiome and change the abundance of N-cycling related microorganisms [27,28]. Thus, organic fertilizers can regulate plant growth and enhance plant nutrient availability, which directly affects crop yields. Organic fertilizers allow the plant to take advantage of both total and available nutrients. This leads to a reduced nutrient leaching loss and improved biological functions [29]. In addition, the application of organic fertilizers reduces the occurrence of soil-borne diseases by promoting the specific plant-beneficial microbial consortia in the reassembled microbial communities [30].

Many studies on the Sanqi ginseng farming system have shown the effect of crop rotation, monoculture, and chemical fertilizer application on soil microbial communities and their relationship with disease development [20,31]. However, the role of bioorganic fertilizers on *P. notoginseng* growth and rhizosphere microbiome is yet to be clarified. In this study, we investigated the impact of an organic fertilizer on soil physicochemical properties, Sanqi ginseng growth, rhizosphere bacterial communities, and metabolites. Two treatments were designed: application of a bioorganic fertilizer in soils planted with Sanqi ginseng and soils receiving no fertilizer. This experimental design allowed us to clarify the relative contribution of the organic fertilizer on *P. notoginseng* growth parameters such as plant height, fresh and dry weight. We tracked the changes in the rhizosphere bacterial communities of Sanqi ginseng grown in fertilized and non-fertilized soils. We also determined the changes in soil metabolites and then correlated them with the bacterial communities. By decrypting the mode of action of the applied bioorganic fertilizer, we sought to determine how bioorganic fertilizers positively affect the Sanqi ginseng growth by modulating the rhizosphere microbiome and metabolites.

## 2. Materials and Methods

### 2.1. Study Site and Sample Collection

The field site is located at Kunming Xundian Undergrowth Planting Base, Yunnan Province, China (22°37′ N, 20°93′ E) at an altitude of 1764 m. The region has a subtropical climate with an annual precipitation of 950–1000 mm and an annual temperature of 18–24 °C. In 2017, a bioorganic fertilizer (Yuesheng brand) obtained from Shanghai Luyuan Three Elements Biological Technology Co., Ltd., Kumming, China, was initially applied to the field at a rate of 3000 kg/hectare. Then, *P. notoginseng* was transplanted into the fertilized soil. The bioorganic fertilizer was used again in 2018 as topdressing on the surface of the planted soil at a rate of 1200 kg/hectare. We ensured that no fertilizer residue was left on the surface of plant leaves. In the same field, a portion without application of the bioorganic fertilizer served as control. The experimental design was a split plot with three replications and two treatments. After two years of *P. notoginseng* plantation, soil samples were collected. We pooled five *P. notoginseng* plants from plot together for the collection of the rhizosphere soil samples. A total of three rhizosphere samples were collected for the bioorganic fertilizer treatment (*n* = 3) and control treatment (*n* = 3) to analyze bacterial communities and metabolites. Bulked soil samples collected around the *P. notoginseng* plants were analyzed for physicochemical properties.

### 2.2. Soil Physicochemical Analysis

The physicochemical properties of the soil samples were quantified as previously reported [32]. Soil pH was analyzed using an FE-20 pH meter (Swiss Mettler). The organic matter (OM) contents were analyzed using the dichromate chemical oxygen demand test. Total nitrogen (TN), total phosphorus (TP), and total potassium (TK) were measured after soil being treated by an H_2_SO_4_-H_2_O_2_ mixture. An autoAnalyser3 (Bran + Luebbe, Hamburg, Germany) was used to determine TN and TP, while TK was measured by a flame atomic spectrophotometry. Alkali-hydrolyzed nitrogen (AN), available phosphorus (AP), and available potassium (AK) were analyzed by the diffusion method, the Olsen method, and the ammonium acetate extraction flame photometry method, respectively.

### 2.3. Rhizosphere Soil Collection

Rhizosphere soils of *P. notoginseng* grown in fertilized and non-fertilized fields were collected using the standard protocol. Briefly, *P. notoginseng* roots were gently shaken to remove bulk soil, and then the roots were transferred to 50 mL Falcon tubes with 25 mL of sterile Silwet L-77 amended PBS buffer. Falcon tubes were continuously rotated on a shaking platform for 20 min at 180 rpm to separate closely adhered soil from the root. The roots were carefully removed after the rhizosphere soil settled down, and the washing buffer was centrifuged at 10,000× *g* for 20 min. The supernatant was discarded, and the resulted rhizosphere soil samples were used for microbiome and metabolites analysis.

### 2.4. Rhizosphere Microbiome Analysis

Rhizosphere soils DNA extracted using the FastDNA Spin Kit for Soil (MP Biomedicals) by following the manufacturer’s guideline. Samples were homogenized in the FastPrep instrument for 40 s at a speed setting of 6.0. The DNA was eluted in 50 µL of elution buffer, and the PCR amplification of the V3-V4 region of 16S rRNA gene carried out using primer pair 338F (5′-ACTCCTACGGGAGGCAGCAG-3′) and 806R (5′-GGACTACHVGGGTWTCTAAT-3′) [33]. PCR amplicons were purified using AMPure XT beads (Beckman Coulter Genomics, Danvers, MA, USA) and quantified using Qubit (Invitrogen, Waltham, MA, USA). Finally, the paired-end sequencing of the bacterial amplicons was performed on the Illumina NovaSeq PE250 platform. Raw bacterial reads were first quality-trimmed using Trimmomatic and then assigned to samples based on barcodes. Chimeric sequences were identified using Vsearch software, and sequences characterized as chimeric were removed. Bacterial sequences were binned into operational taxonomic units (OTUs) at ≥97% similarity level through open-reference OTU picking protocol in the UPARSE-pipeline. The most abundant sequences from each OTU were taken as representative sequences for the respective OTU. Taxonomic configuration of OTUs performed using the Silva database.

### 2.5. Metabolites Profiling from Rhizosphere Samples

The soil samples were homogenized in a mixer mill (MM 400, Retsch, Hann, Germany) with a zirconia bead for 1.5 min at 30 Hz. A 100 mg sample from each replicate was weighted and extracted overnight at 4 °C with 1.2 mL 70% aqueous methanol. The extracts were filtrated after centrifugation at 12,000 rpm for 10 min for ultra-performance liquid chromatography-tandem mass spectrometry (UPLC-MS/MS) analysis and analyzed using a UPLC-ESI-MS/MS system.

### 2.6. Statistical Analysis and Data Visualization

We used QIIME to calculate the alpha diversity, including the observed species and Shannon diversity index, and visualized the values in the boxplot using R-3.5.3. The weighted unifrac distance for beta diversity analysis was also calculated using QIIME, and performed the Principal Coordinate Analysis (PCoA) to get principal coordinates and visualize multidimensional data in R package ggplot2. Weighted Unifrac distance data was also used to perform UPGMA clustering of fertilized and non-fertilized soil samples. The relative abundances of the bacteria at different taxonomic levels were calculated based on the classified OTU reads and were subsequently plotted in R with the package ggplot2. Differences between bioorganic fertilizer and non-fertilizer treatments were calculated using a Welch’s t-test.

Identified metabolites were annotated using the Kyoto Encyclopedia of Genes and Genomes (KEGG) Compound database (http://www.kegg.jp/kegg/compound/, accessed on 10 August 2020), annotated metabolites then mapped to KEGG Pathway database (http://www.kegg.jp/kegg/pathway.html, accessed on 10 August 2020). Pathways with significantly regulated metabolites mapped to were then fed into metabolite sets enrichment analysis; their significance was determined by hypergeometric test’s p-values. The hierarchical cluster analysis (HCA) results of samples and metabolites were presented as heatmaps with dendrograms. In contrast, Pearson correlation coefficients (PCC) between samples were calculated by the cor function in R and presented as only heatmaps. Both HCA and PCC carried out by R package pheatmap. Significantly regulated metabolites between groups were determined by variable importance in projection, VIP ≥ 1 and absolute Log2 FC (fold change) ≥1. VIP values were extracted from the Orthogonal partial least squares discriminant analysis (OPLS-DA) result, which contains score plots and permutation plots, and was generated using R package MetaboAnalystR. The data was log transform (log2) and mean centering before OPLS-DA. To avoid over fitting, a permutation test (200 permutations) was performed. Spearman’s correlation index values among the specific microbial taxa and metabolites were calculated using the “psych” package in R 3.4.0, and we removed the correlations with a Spearman’s coefficient < 0.7 and *p* > 0.05.

## 3. Results

### 3.1. Impact of a Bioorganic Fertilizer Application on Sanqi Ginseng Growth and Soil Physicochemical Properties

**Figure 1:**
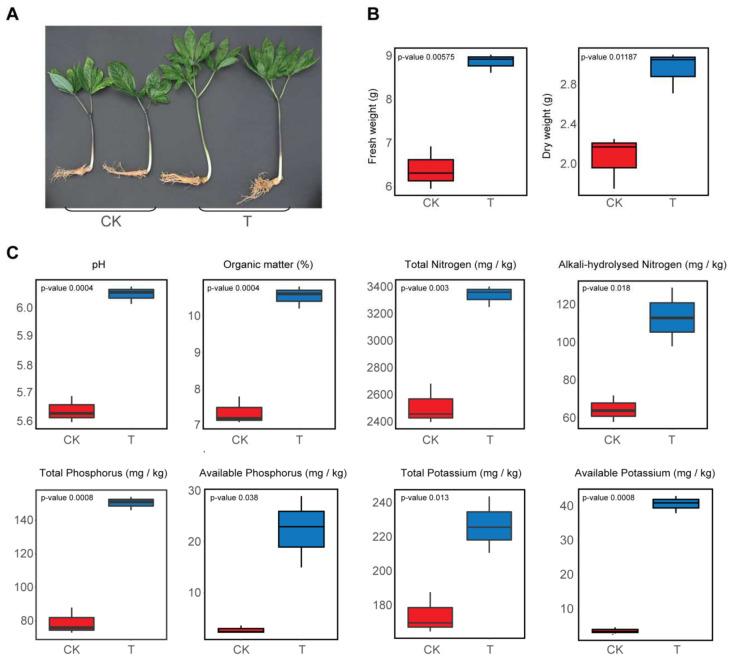
Effect of a bioorganic fertilizer application on *P. notoginseng* fresh and dry weights, root and shoot lengths, and soil physicochemical properties. (**A**) Representative images of *P. notoginseng* plants grown in soils without (CK) and with (T) a bioorganic fertilizer. (**B**) *P. notoginseng* plants fresh weight, dry weight, shoot length and root length. (**C**) Comparison of soil physicochemical properties between bioorganic fertilizer and non-fertilizer treatments. Whiskers in the red and blue box represent the range of minimum and maximum values within a control and bioorganic fertilizer treatment, respectively.

After two years of *P. notoginseng* cultivation, the bioorganic fertilizer treatment produced taller plants than the untreated control (Figure 1A). Plant fresh weight (FW) and dry weight (DW) were significantly increased in plots that received the bioorganic fertilizer (FW, 8.85 ± 0.12 g; DW, 2.95 ± 0.12 g) compared to non-fertilizer (FW, 6.39 ± 0.28 g; DW, 2.05 ± 0.16 g) treatment (Figure 1B). Notably, the soil pH was alleviated to 6.04 ± 0.030 from 5.64 ± 0.020, and the soil moisture was reduced to 17.22 ± 0.001 from 26.42 ± 0.002 by the addition of the bioorganic fertilizer (Figure 1C). The soil total organic matter (TOM) is an essential indicator of soil health. Compared with plots that received no fertilization, the TOM contents significantly increased in soils after addition of the bioorganic fertilizer. Similarly, the TN, AN, TP, AP, TK and AK contents were also improved in soils amended with the bioorganic fertilizer. Overall, the bioorganic fertilizer improved Sanqi ginseng plant growth, alleviated soil acidification and increased essential nutrient contents in the soil.

### 3.2. The Bioorganic Fertilizer Alters P. notoginseng Rhizosphere Bacterial α- and β-Diversity

**Figure 2:**
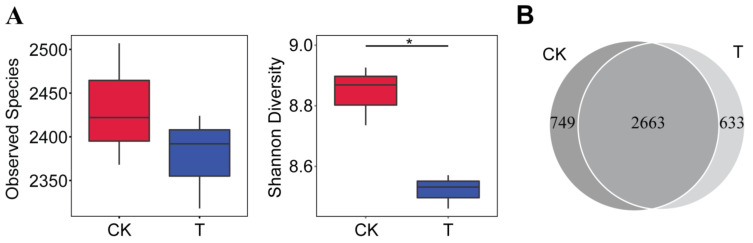
Bioorganic fertilizer alters bacterial community alpha diversity. (**A**) Alpha diversity of rhizosphere bacterial community in the bioorganic fertilizer (T) and no-fertilizer (CK) treatments. Whiskers in the red and blue box represent the range of minimum and maximum alpha diversity values within a control (CK) and bioorganic fertilizer (T) treatment.The bacterial diversity values were considered significantly different between treatments when the *p*-value < 0.05 (* *p* < 0.05). (**B**) Venn diagram of OTUs detected in the bioorganic fertilizer and no-fertilizer treatments.

The impacts of the bioorganic fertilizer on the rhizosphere bacterial community richness and diversity are shown in Figure 2. The boxplots based on the observed species showed that the addition of the bioorganic fertilizer reduced the number of bacterial taxa in the rhizosphere, but the values were not different compared to the mock treatment. In contrast, the Shannon diversity values were significantly decreased for the rhizosphere bacterial community of Sanqi ginseng grown in soils amended with the bioorganic fertilizer relative to the non-fertilizer treatment. This observation points out that the bioorganic fertilizer alters the α-diversity of the rhizosphere microbiome (Figure 2A). Among the total bacterial OTUs detected from both treatments, 633 OTUs were unique to the bioorganic fertilizer treatment, and 749 OTUs were unique to the non-fertilizer treatment. A total of 2663 OTUs were shared between the two groups (Figure 2B).

**Figure 3:**
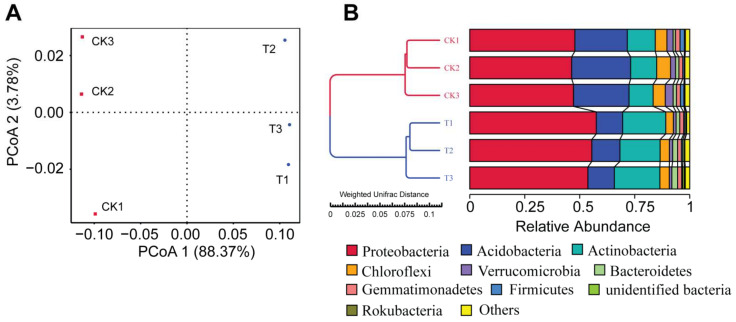
Bioorganic fertilizer alters bacterial community beta diversity. (**A**) PCoA analysis based on weighted UniFrac distance of rhizosphere bacterial communities at the phylum level in the bioorganic fertilizer and no-fertilizer treatments. (**B**) The UPGMA tree is showing clusters of bacterial communities at the phylum level based on weighted UniFrac distance.

Next, we performed a principal coordinate analysis (PCoA) based on the weighted UniFrac distance to observe changes in the rhizosphere bacterial community structure (Figure 3A). The bacterial community inhabiting the rhizosphere of *P. notoginseng* grown in the bioorganic fertilizer amended soils was clearly separated from the control treatment. Both treatments separated along the axis 1, and the first coordinate of PCoA 1 explained a maximum variation of 88.37% in the bacterial β-diversity. The observed differences in β-diversity were mainly explained by the change in the proportion of dominant bacterial phyla inhabiting the rhizosphere (Figure 3B). These results indicate that the application of the bioorganic fertilizer had a significant effect on the rhizosphere community.

### 3.3. Effect of a Bioorganic Fertilizer Application on Rhizosphere Bacterial Community Composition

**Figure 4:**
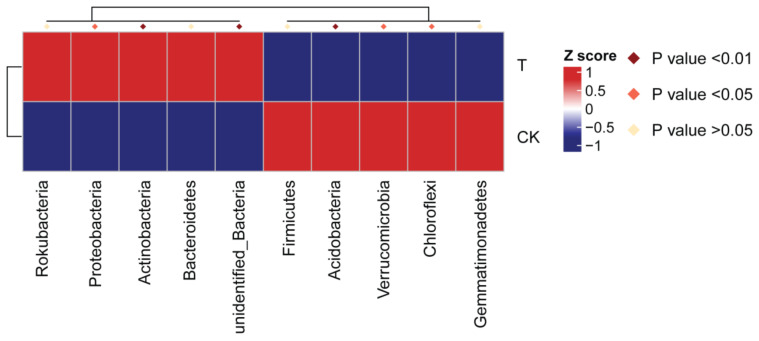
Relative abundance of bacterial phyla found to be significantly different between the bioorganic fertilizer and no-fertilizer treatments. The bacterial phyla composition was considered significantly different between treatments when the *p*-value was <0.05.

A total of 386 bacterial genera belonging to 38 phyla were identified in the rhizosphere of *P. notoginseng*. Among them, only eight phyla and 11 genera have relative abundances greater than 1%. At the phylum level, Proteobacteria (51.3%), Acidobacteria (18.7%), and Actinobacteria (15.6%) dominated the rhizosphere bacterial community. Specifically, the phyla Proteobacteria, Acidobacteria, Actinobacteria, Chloroflexi, and Verrucomicrobia were found to be significantly different in relative abundance between the bioorganic fertilizer and control treatments (Figure 4).

**Figure 5:**
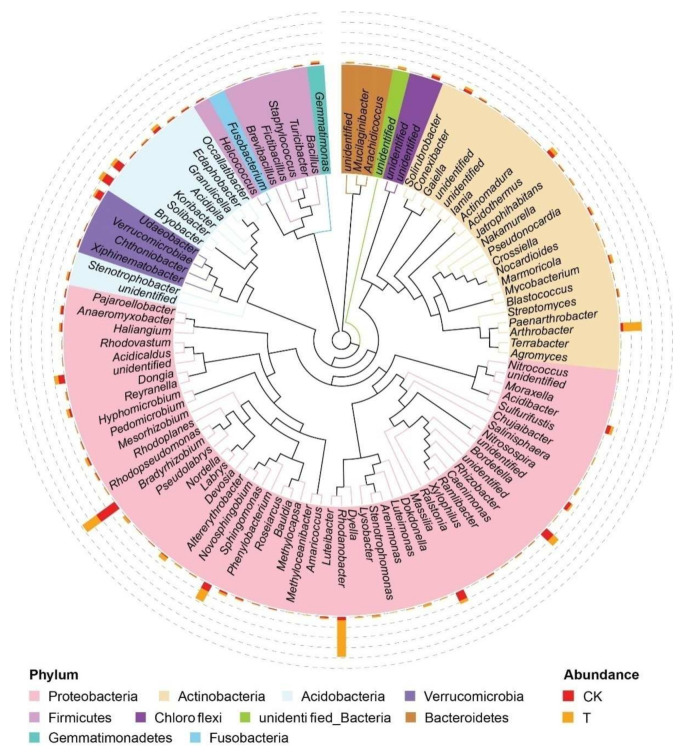
Phylogenetic evolutionary tree of rhizosphere bacterial microbiota at the genus level from the bioorganic fertilizer and no-fertilizer treatments. Different colors of the branches represent different phyla. The relative abundance of each genus displayed outside the circle with orange (T, bioorganic fertilizer) and red color (CK, no-fertilizer).

The relative abundance of Proteobacteria and Actinobacteria increased, but Acidobacteria, Chloroflexi and Verrucomicrobia decreased in abundance with the bioorganic fertilizer application. At the family level, the relative abundance of Rhodanobacteraceae, Micrococcaceae, Sphingomonadaceae, and Micropepsaceae increased in the bioorganic fertilizer treatment (Figure S1). A phylogenetic evolutionary tree was constructed to represent the dominant phyla and genera (Figure 5).

**Figure 6:**
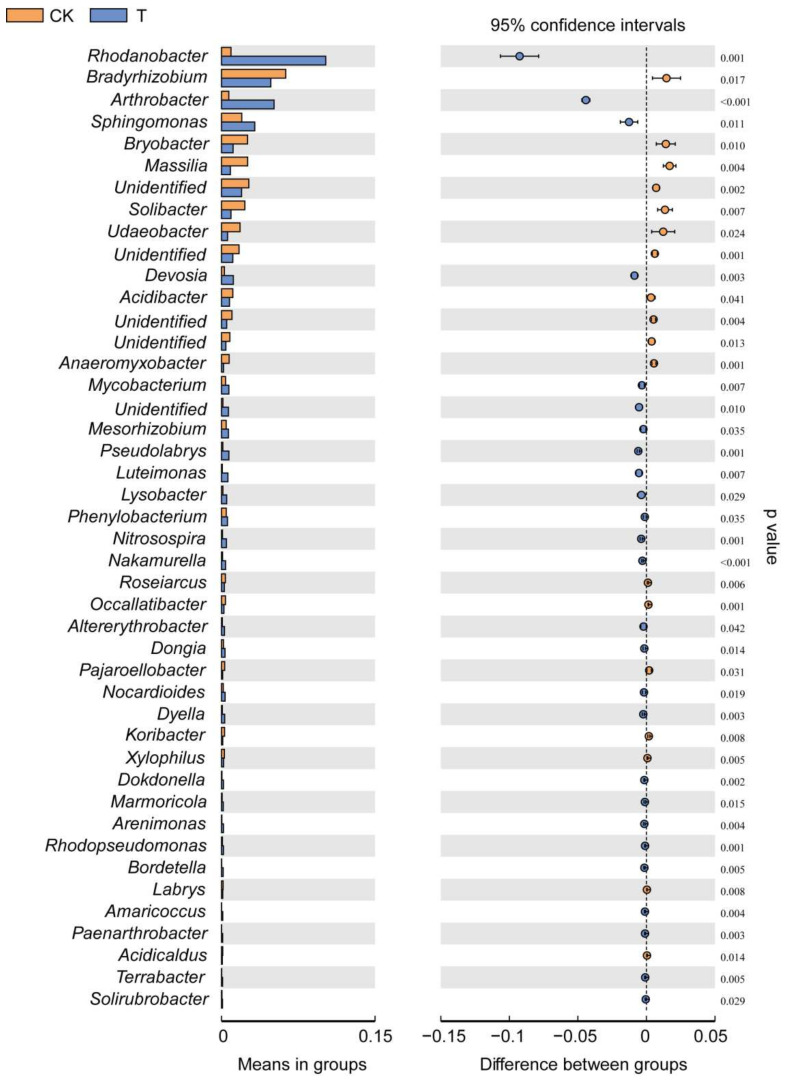
Extended error bar plot indicates the differences in bacterial genera relative abundance in the bioorganic fertilizer and no-fertilizer treatments. Only significantly different genera between the two groups were depicted (Welch’s *t*-test, *p* < 0.05).

Covering the phylum to genus level, the genera that significantly changed in abundance after adding the bioorganic fertilizer were identified using the Welch’s t-test. The relative abundance of several bacterial genera including, *Rhodanobacter*, *Arthrobacter*, *Sphingomonas*, *Devosia*, *Pseudolabrys*, *Luteimonas*, *Lysobacter*, *Nitrosospira* and *Nakamurella* increased in the rhizosphere of Sanqi ginseng grown in soils amended with the bioorganic fertilizer. In contrast, the abundance of bacterial genera *Bradyrhizobium*, *Bryobacter*, *Massilia*, *Solibacter*, and *Udaeobacter* increased in the non-fertilizer treatment (Figure 6). Most of the bacterial genera abundant in the bioorganic fertilizer treatment were also positively correlated with the soil physicochemical properties (Figure S2).

### 3.4. Changes in Soil Metabolites after Addition of the Bioorganic Fertilizer

**Figure 7:**
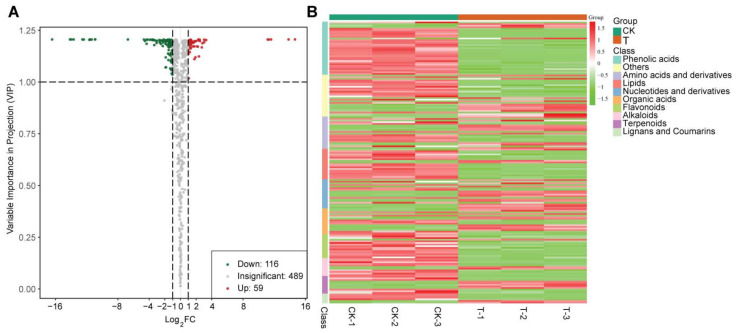
Differentially abundant soil metabolites in bioorganic (T) and no-fertilizer (CK) treatments. (**A**) Volcano plot showing numbers of significantly enriched metabolites in bioorganic fertilizer (red circles) and no-fertilizer (green circles) treatments. (**B**) Heat map representing various metabolites classes detected from bioorganic and no-fertilizer treatments.

Soil metabolomics was performed in order to determine the bacterial microbiota responses to the application of the bioorganic fertilizer. Of the 664 metabolites detected by the UPLC-ESI-MS/MS, 175 metabolites were found to be differentially accumulated, and among them, 59 metabolites were enriched in soils amended with the bioorganic fertilizer (Figure 7A). A heatmap was constructed to show the differences in the abundance of various metabolites assigned to several classes. Metabolites mainly belong to the classes of phenolic acids, lignans and coumarins, amino acids and derivatives, lipids, nucleotides and derivatives, organic acids, flavonoids, alkaloids, and terpenoids (Figure 7B). Many compounds in the class of phenolic acids, flavonoids, lipids and alkaloids were significantly enriched in the non-fertilizer treatment and decreased in composition after applying the bioorganic fertilizer.

Specifically, D-Threitol, O-Phospho-L-serine, Nicotianamine, 3-Aminosalicylic acid, Ribulose-5-phosphate, Uridine 5′-diphospho-D-glucose, 3′-Dephospho-CoA, Guanosine 3′,5′-cyclic monophosphate, S-(Methyl)glutathione, and Xanthine were the predominantly enriched metabolites in the soil amended with bioorganic fertilizer. In contrast, Maleoyl-caffeoylquinic acid, 3′-Methoxydaidzin, Sodium ferulate, Asperulosidic acid, p-Coumaric acid, Methylenesuccinic acid, 2-(Formylamino)benzoic acid, Petunidin-3-O-glucoside-5-O-arabinoside, Esculetin, and Kaempferol-3-O-(2″-O-acetyl)glucuronide were the predominantly enriched metabolites in the non-fertilizer treatment (Figure S3). These differentially abundant metabolites were further found to positively and negatively correlate with the soil physicochemical properties in the bioorganic fertilizer and non-fertilizer treatments, respectively (Figure S4).

### 3.5. Correlations between the Bacterial Community and Soil Metabolism

**Figure 8:**
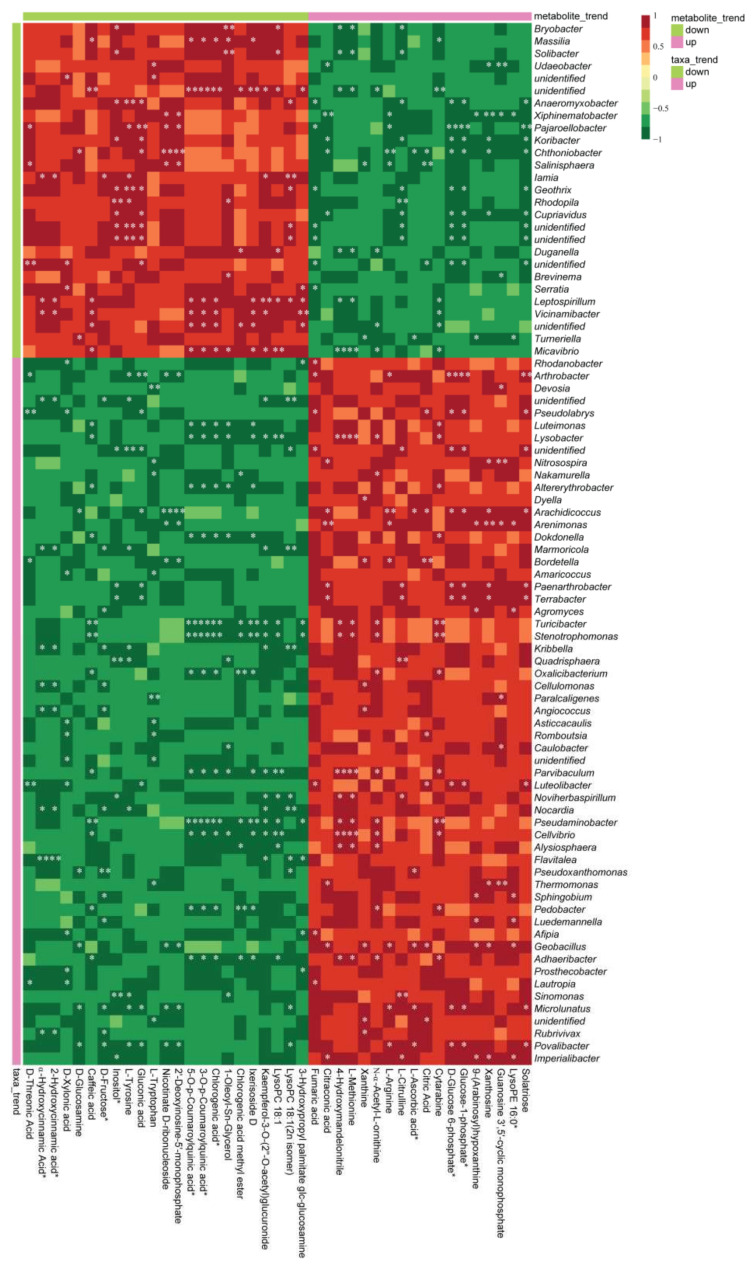
Heatmap based on Spearman’s correlation index values between differential microbial taxa and metabolites responding to bioorganic fertilizer and no fertilizer treatments in the rhizosphere. Correlation were considered significant when the *p*-value < 0.05 (* *p* < 0.05, ** *p* < 0.01).

Soil microorganisms are one of the crucial drivers of the distribution of metabolites. We performed a Spearman’s correlation and constructed a co-occurrence network and heatmap to show the relationship between the differential metabolites and significantly impacted bacterial taxa (Figure 8 and Figure S5).

Several bacterial genera were positively correlated with soil metabolites in the bioorganic fertilizer and non-fertilizer treatments. For the bioorganic fertilizer effect, the soil metabolites that were correlated with enriched genera included those from the class of *organic acids* (citraconic acid and fumaric acid, citric acid); *saccharides and alcohols* (Solatriose, Glucose-1-phosphate, D-Glucose 6-Phosphate); *lipids* (LysoPE 16:0); *nucleotides and derivatives* (Guanosine 3′, 5′-cyclic monophosphate, Xanthosine, 9-(Arabinosyl)hypoxanthine, Cytarabine, Xanthine); *vitamin* (L-Ascorbic acid), *amino acids and derivatives* (L-Citrulline, L-Arginine, N-α-Acetyl-L-ornithine, L-Methionine); and *alkaloids* (4-Hydroxymandelonitrile). A genus *Rhodanobacter* was highly enriched in the rhizosphere bacterial community of Sanqi ginseng and positively correlated with Fumaric acid (Figure 8). The second highly enriched genus in the rhizosphere was *Arthrobacter* and was positively correlated with the metabolites such as Solatriose, Glucose-1-phosphate, D-Glucose 6-Phosphate, L-Arginine, and Fumaric acid. Except for *Arthrobacter*, Glucose-1-phosphate and D-Glucose 6-Phosphate were also positively correlated with the genera *Paenarthrobacter*, *Terrabacter*, *Luteolibacter*, and *Povalibacter* (Figure 8). In contrast, phenolic acids correlated with bacteria enriched in the non-fertilizer treatment. For example, α-Hydroxycinnamic Acid, 2-Hydroxycinnamic acid, Caffeic acid, 5-O-p-Coumaroylquinic acid, 3-O-p-Coumaroylquinic acid, and Chlorogenic acid methyl ester positively correlated with several bacterial taxa abundant in the soil without the bioorganic fertilizer. Especially, a positive correlation between the genus *Massilia* and 5-O-p-Coumaroylquinic acid, 3-O-p-Coumaroylquinic acid, and Caffeic acid was detected (Figure 8).

## 4. Discussion

Excessive uses of chemical fertilizers and pesticides have caused a deterioration of soil health and microbiome communities. Hence, replacing the inorganic with bioorganic fertilizers is a prerequisite for sustainable agriculture [13]. Previous studies have shown that applying organic fertilizers improves soil and plant health and contributes to high crop yield [12]. In this study, we investigated the effect of a bioorganic fertilizer on the soil physicochemical characteristics, soil metabolites, Sanqi ginseng growth, and rhizosphere microbiome. Compared to the no-fertilizer treatment, the bioorganic fertilizer application improved Sanqi ginseng plant fresh weight, dry weight, shoot length, and root length. Soil organic matter, total and alkali-hydrolyzednitrogen, and total and available phosphorus were also higher in soil amended with bioorganic fertilizer. Generally, the increased nutrient level in soils has been linked with improved plant performance. High amounts of available nitrogen, phosphorus and potassium in soil improve crop quality and yield [34]. It has been previously observed that the amount of organic matter in soil is directly proportional to the yield production [35]. Moreover, the addition of inorganic fertilizers does not affect total nitrogen content, but the organic fertilizers are confirmed to improve soil nutrients for maintaining stable yields [36,37,38]. Amendment of excessive inorganic nitrogen fertilizers is also hypothesized to cause soil acidification because of soil nitrification [39,40,41]. In our study, the application of a bioorganic fertilizer altered soil pH from 5.64 to 6.04, thus reduced the soil acidification. Previously, Zhang et al. [42] demonstrated that applying a high proportion of organic fertilizers relative to a low proportion alleviates soil acidification. These results highlight that the soil amendment of organic fertilizers improves soil nutrient and organic matter and reduces soil acidification associated with high plant performance.

Microorganisms are an important component of soil ecosystems that is directly associated with plant health [43]. Organic and inorganic amendments significantly affect the soil microbiome structure and functions. In our study, the addition of a bioorganic fertilizer affected the bacterial diversity. Shannon diversity decreased, and bacterial community composition shifted in the bioorganic fertilizer treatment compared to non-fertilizer treatment. However, the species richness was not significantly different between both treatments. A decrease in bacterial Shannon diversity might be due to plant response to changes in soil environmental conditions caused by bioorganic fertilization leading to the enrichment of a specific subset of functional microbiota in the rhizosphere. Application of the bioorganic fertilizer enriched bacterial phyla Proteobacteria and Actinobacteria and decreased the abundance of phylum Acidobacteria. Some of the members of Proteobacteria and Actinobacteria are copiotrophic bacteria [44], while some members of Acidobacteria are fastidious oligotrophic bacteria [44]. The increased relative abundance of some of the members of Proteobacteria in the rhizosphere has been positively correlated with the increased soil nutrient level [45]. Several bacterial species within phylum Proteobacteria dominate the nutrient-rich environment and play a key role in C and N cycling [46]. Similarly, increased availability of C and N is also known to induce the abundance of some of the members of Actinobacteria in the soil [47]. For Acidobacteria, the low soil pH increased its relative abundance in the soil as some bacterial taxa in this phylum are often negatively correlated with the soil pH [48,49]. This phenomenon highlights the general life-history strategies that addition of nutrients favoring the fast growing and copiotrophic bacteria [50]. Overall, these results suggest that the addition of a bioorganic fertilizer is likely to favor the growth of some copiotrophic bacteria (e.g., some bacteria within phylum Proteobacteria and Actinobacteria) over some oligotrophic bacteria (e.g., some bacteria within phylum Acidobacteria) because of their ability to live in nutrient-sufficient or nutrient-limited environments, respectively.

Soils from the bioorganic fertilization treatment are enriched in several bacterial genera, including *Rhodanobacter*, *Arthrobacter*, *Sphingomonas*, *Devosia*, *Pseudolabrys*, *Luteimonas*, *Lysobacter*, *Nitrosospira* and *Nakamurella.* Bacterial species in the genus *Rhodanobacter* are Gram-negative, rod-shaped and aerobic, mainly catalase- and oxidase-positive [51]. *Rhodanobacter* was previously positively correlated with the soil pH and negatively with the soil NO_3_^−^N [52]. In this study, *Rhodanobacter* was also positively correlated with the soil pH and other soil chemical properties. Interestingly, *Rhodanobacter* and *Lysobacter* were also found as antagonistic to fungal pathogens in previous reports [53,54]. *Lysobacter* was not significantly correlated with soil properties in the present study and, therefore, might be involved in functions for the plants other than nutrient acquisition. The abundance of some *Arthrobacter* species was increased with the addition of the bioorganic fertilizer [55] and it has been found that they promote plant growth [56] and also restrain pathogenic bacteria and fungi [57]. *Sphingomonas* is ubiquitous in natural habitats and has been reported to be involved in disease suppression [58,59,60]. *Devosia* sp. was previously found to form a unique nitrogen-fixing foot-nodule symbiosis with the aquatic legume *Neptunia natans* (Lf) Druce [61] and has been correlated with nitrogen in the soil [62]. With reference to these studies, we deduce that *Devosia* sp. increase available nitrogen contents in soil. The genus *Pseudolabrys* was previously reported to increase in relative abundance after NK treatment [63]. *Luteimonas* species are known for their catalytic activities related to oxidase, catalase, alkaline phosphatase, esterase, and esterase lipase and are involved in the biodegradation metabolism of the organic matter [64,65,66]. The enrichment of *Luteimonas* in soil is a valuable indicator of soil amelioration [67]. The relative abundance of *Nitrosospira* was positively correlated with nitrification activity following long-term inorganic and organic fertilization [68]. Most of the bacterial genera enriched in the rhizosphere of *P. notoginseng* due to the addition of the bioorganic fertilizer were positively correlated with soil properties such as pH, TOM, TN, AN, TP, AP, TK, and AK. In contrast, *P. notoginseng* grown in soil without fertilizer was enriched for bacterial genera *Bradyrhizobium*, *Bryobacter*, *Massilia*, *Solibacter*, *Udaeobacter*, and *Acidibacter*. *Bradyrhizobium* spp. are agriculturally important because they can form root nodules and be involved in nitrogen fixation.

Soil metabolites mainly source from microbial metabolites, soil organic matter, plant decomposition, and root exudates [69]. In rhizosphere metabolomics, differentiating the metabolites from plants and microorganisms is still a big challenge [70]. Soil microorganisms mainly control most of the reactions in the N cycle [71,72,73]; therefore, an intense interaction may occur between bacterial microbiota after the addition of bioorganic fertilizers. In this study, the addition of a bioorganic fertilizer altered the metabolomic profile of rhizosphere soils and decreased the composition of many compounds belonging to the classes of phenolic acids, flavonoids, lipids, and alkaloids. We observed that the p-Coumaric acid was the predominantly enriched metabolite in the non-fertilizer treatment, and its abundance decreased after adding the bioorganic fertilizer. Previous studies have demonstrated that accumulation of phenolic acids in the soil inhibits seedling growth [74], especially p-Coumaric acid, which may inhibit plant growth [75]. Furthermore, we found a significant and positive correlation between the genus *Massilia* and 5-O-p-Coumaroylquinic acid, 3-O-p-Coumaroylquinic acid, and caffeic acid in the non-fertilizer treatment. Previous studies found that the presence of pathogens increased the release of caffeic acid and that the caffeic acid and infected plant exudates have reasonably similar effects on microbial community composition [76]. The enrichment of caffeic acid indicates that the Sanqi ginseng plants in the non-fertilizer treatment were probably under attack by the soil-borne pathogens. Flavonoids contents are found to be related to available nitrogen [77]. Plants accumulate more flavonoids under limiting nitrogen availability compared to those that are well supplied [78]. The metabolites correlated with enriched genera for the bioorganic fertilizer effect included those from the class of organic acids, saccharides and alcohols, and amino acids and derivatives. As organic acids, saccharides and alcohols, and amino acids were enriched in the bioorganic fertilizer treatment; therefore, we focused on deciphering their ecological role in plant–microbe interactions. Organic acids act as chemoattractant signals to microbes and are important nutrients and chelators of poorly soluble mineral nutrients [79,80,81]. Similarly, sugars and sugar alcohols have been shown to act as chemotaxis substances for a range of microbiota in the rhizosphere [79,82]. Zhu et al. [83] reported an increased secretion of sugars and sugar alcohols in the rhizosphere when nitrogen was sufficiently available to the plants. The high composition of amino acids in soil amended with the bioorganic fertilizer indicates that the Sanqi ginseng plants had sufficient nitrogen for growth. It has been documented that plants release the low amount of amino acids when subjected to nitrogen deficiency [84,85]. The above results pointed out the role of bioorganic fertilizers in nutrient availability, disease protection, soil microbial ecology, and crop productivity.

## 5. Conclusions

This study demonstrates that applying bioorganic fertilizers improves the Sanqi ginseng plant growth. We highlight the importance of the rhizosphere bacterial microbiota and their combined functions for nutrient availability and disease protection. The application of the bioorganic fertilizer significantly altered the rhizosphere microbiome and stimulated specific plant-beneficial bacterial consortia, which are involved in nutrient cycling and pathogen suppression. Moreover, the soil bioorganic fertilizer’s addition regulated the correlation between rhizosphere bacterial microbiota and soil metabolism, affecting plant rhizosphere microecology. We recommend using bioorganic fertilizers in Sanqi ginseng production, considering the initial soil nutrient level to ensure a sufficient optimum supply of essential nutrients to plants and stimulate indigenous soil microbiota for natural disease suppression.

## Data Availability

The raw sequencing data has been submitted to NCBI SRA PRJNA752500. All remaining data used in this manuscript are available in the text and additional files.

## Supplementary Materials

The following supporting information can be downloaded at: https://www.mdpi.com/article/10.3390/microorganisms10020275/s1, Figure S1: Relative abundance of bacterial families found significantly different between bioorganic fertilizer and no-fertilizer treatments. The bacterial family composition was considered statistically significant differences when *p* value was less than 0.05; Figure S2: Pearson correlation analysis between significantly enriched bacterial genera (abundant in bioorganic and non-fertilizer treatment) and soil physicochemical properties. The non-significant correlations is shown with cross mark; Figure S3: Differentially abundant top fold change metabolic compounds in bioorganic (red bar) and no-fertilizer (green bar) treatments; Figure S4: Pearson correlation analysis between differentially abundant top fold change metabolic compounds (abundant in bioorganic and no-fertilizer treatments) and soil physicochemical properties. The non-significant correlations is shown with cross mark; Figure S5: Co-occurrence network between differentially abundant bacterial genera and differential metabolites. The red line segment represents positive correlation. The green line segment represents negative correlation. The size of circle represents relative abundance or metabolite expression of bacterial genera.
