# Supplementary material for: Application of Bioorganic Fertilizer on Panax notoginseng Improves Plant Growth by Altering the Rhizosphere Microbiome Structure and Metabolism
Source: Microorganisms. 2022 Jan 25;10(2):275. doi: 10.3390/microorganisms10020275 (PMC8879206; doi:10.3390/microorganisms10020275)
Supplement: Supplementary file 1 [file microorganisms-10-00275-s001.zip › microorganisms-1573476-supplementary.pdf]

Supplementary Information

Figure S1

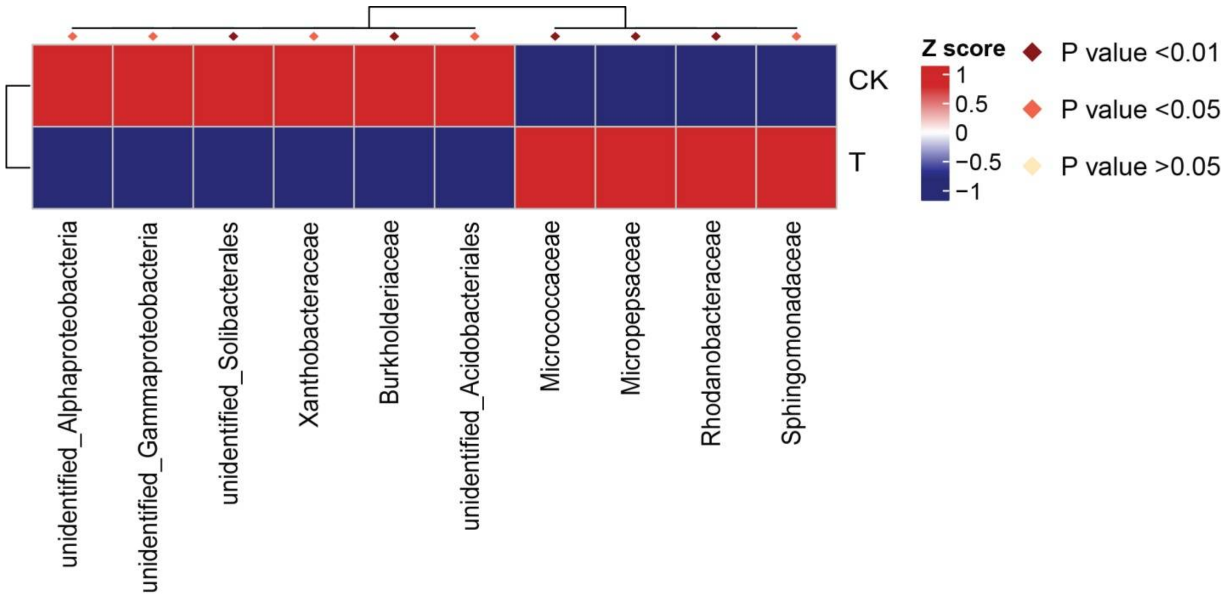

Figure S1. Relative abundance of bacterial families found significantly different between bioorganic fertilizer and no-fertilizer treatments. The bacterial family composition was considered statistically significant differences when p value was less than 0.05.

Figure S2

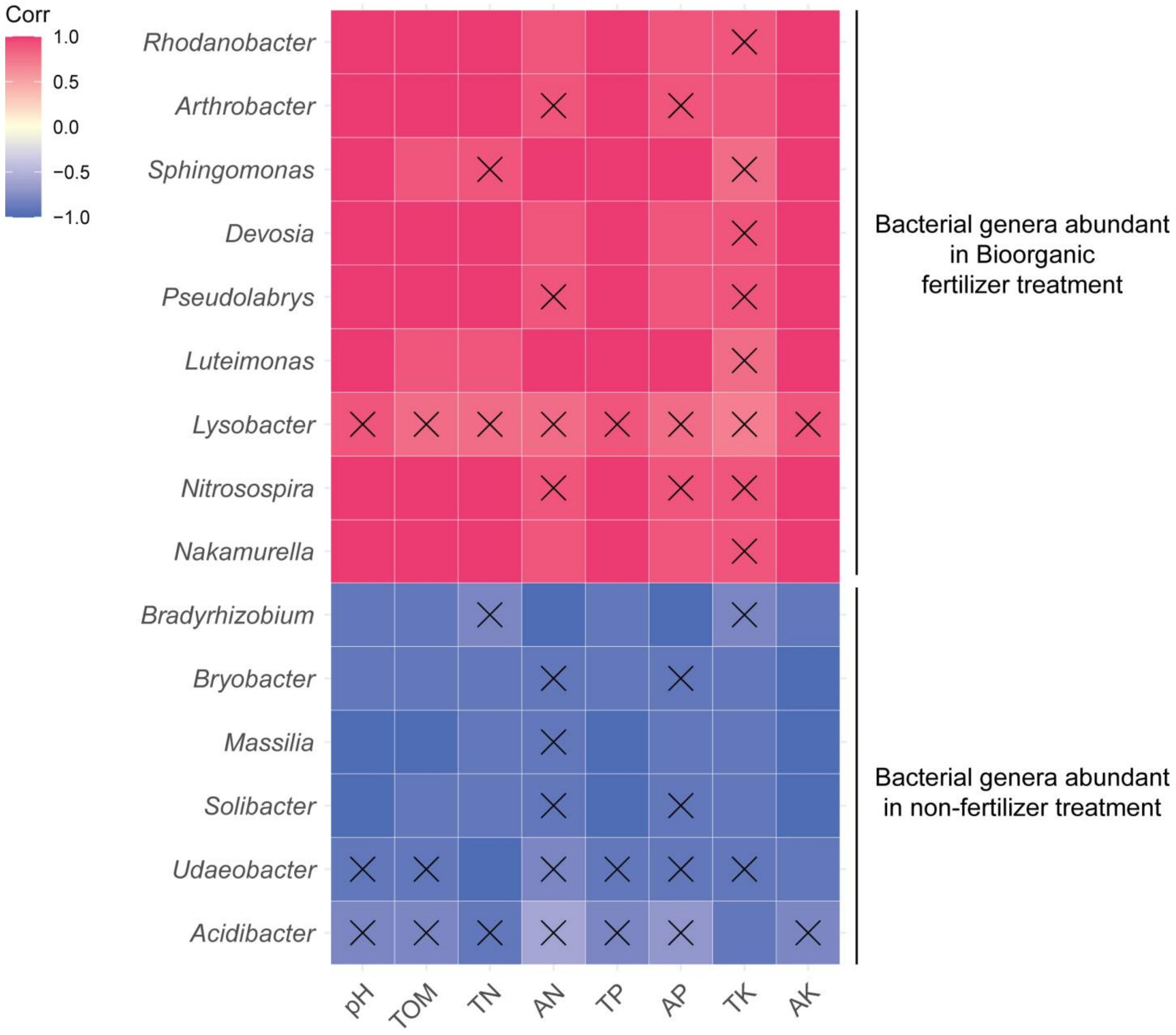

Figure S2. Pearson correlation analysis between significantly enriched bacterial genera (abundant in bioorganic and non-fertilizer treatment) and soil physicochemical properties. The non-significant correlations is shown with cross mark.

Figure S3

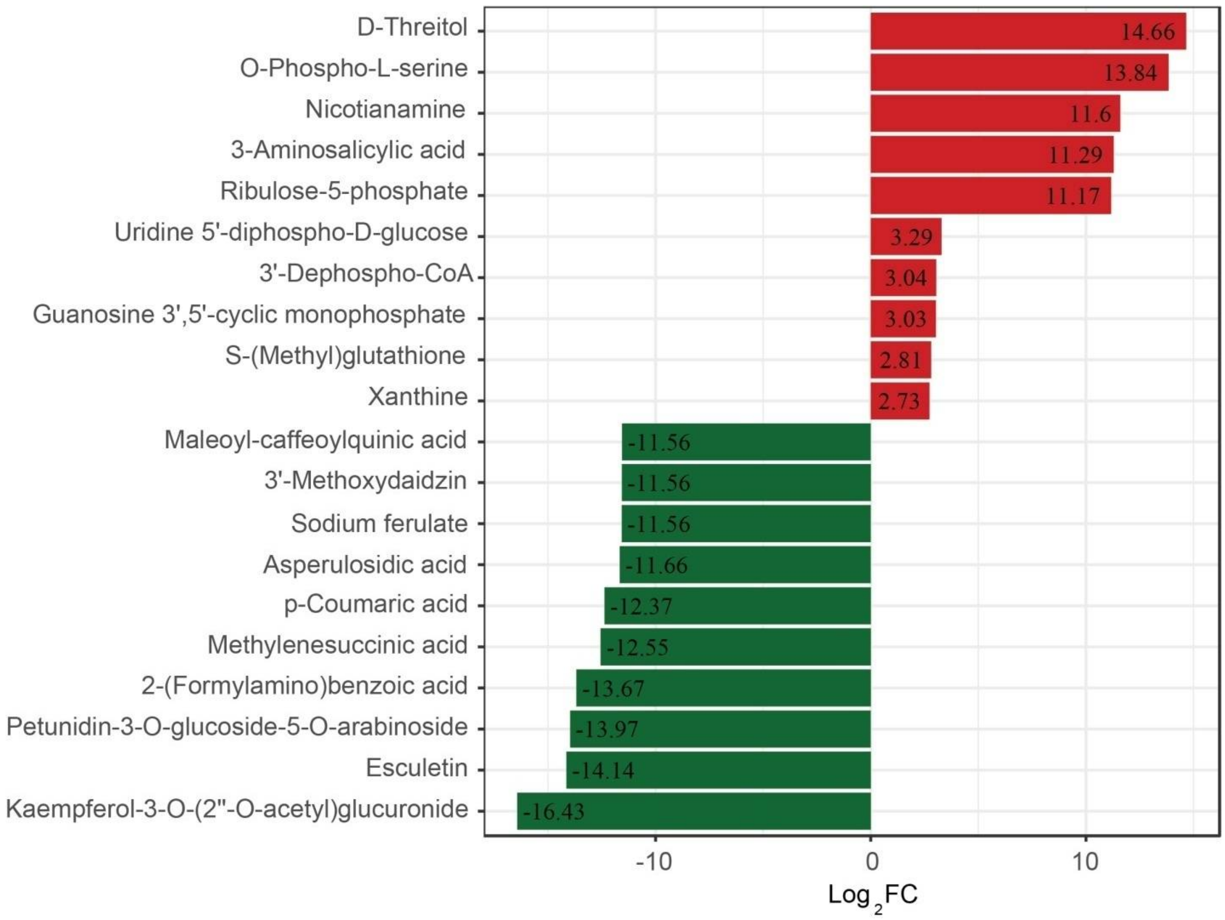

Figure S3. Differentially abundant top fold change metabolic compounds in bioorganic (red bar) and no-fertilizer (green bar) treatments.

Figure S4

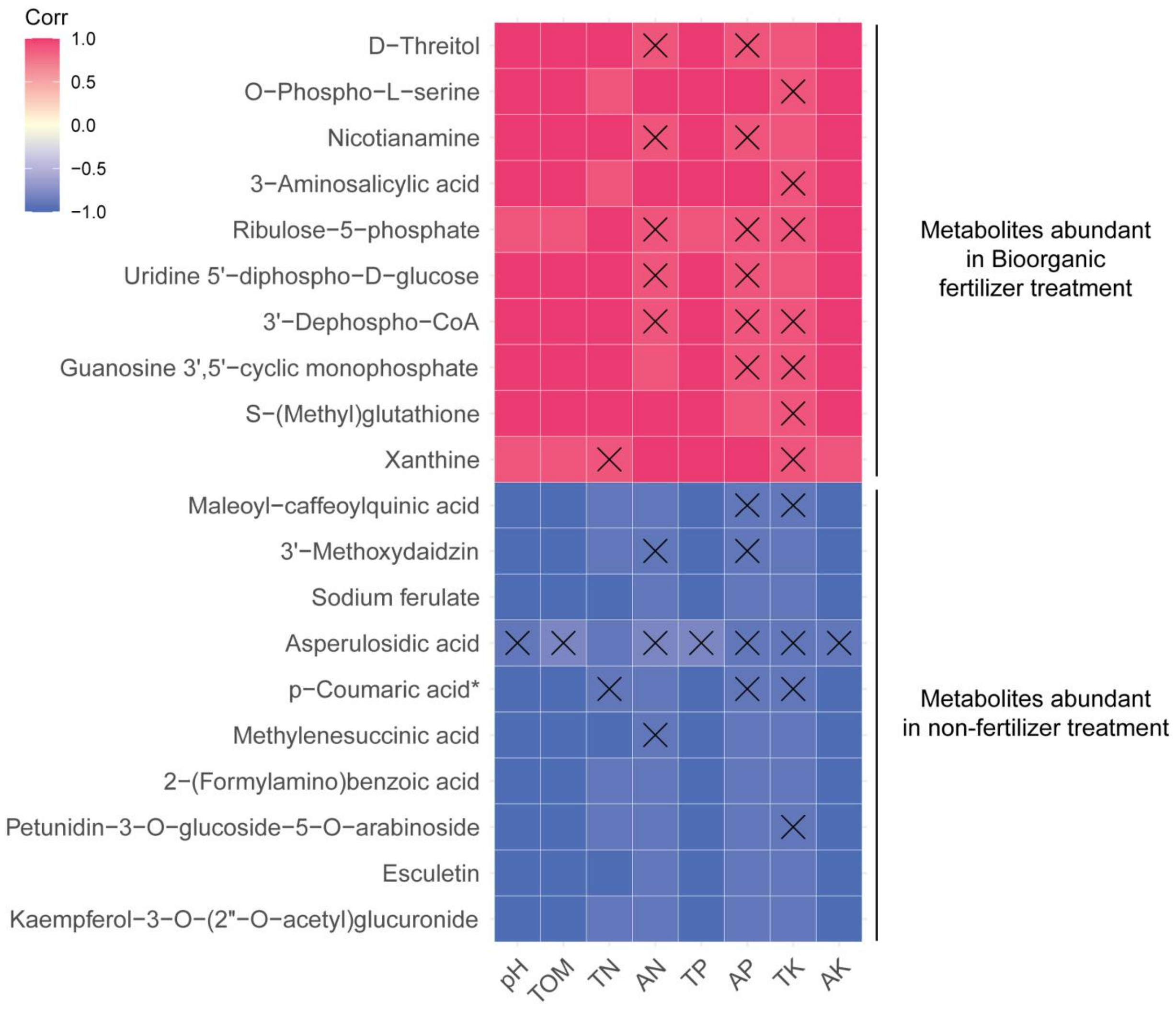

Figure S4. Pearson correlation analysis between differentially abundant top fold change metabolic compounds (abundant in bioorganic and no-fertilizer treatments) and soil physicochemical properties. The non-significant correlations is shown with cross mark.

Figure S5

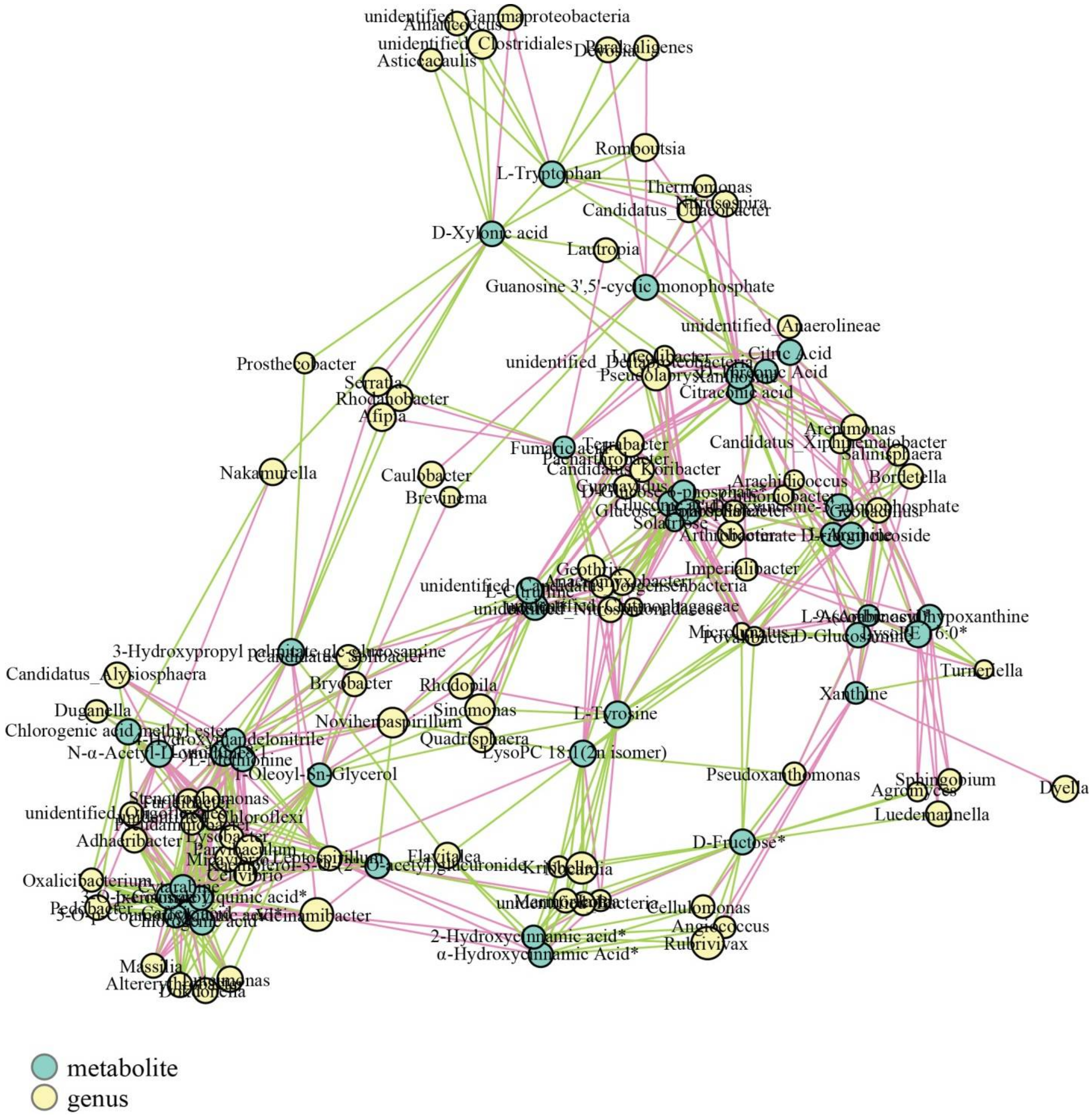

Figure S5. Co-occurrence network between differentially abundant bacterial genera and differential metabolites. The red line segment represents positive correlation. The green line segment represents negative correlation. The size of circle represents relative abundance or metabolite expression of bacterial genera.
